# Advances in Molecular Pathway-Directed Cancer Systems Imaging and Therapy

**DOI:** 10.1155/2014/639475

**Published:** 2014-12-21

**Authors:** David J. Yang, Lan Pham, Mei-Hsiu Liao, Fan-Lin Kong, Hiroji Uemura, Yen-Yu Ian Shih

**Affiliations:** ^1^University of Texas MD Anderson Cancer Center, Houston, TX 77030, USA; ^2^Radiopharmaceuticals Production and Marketing Center, Institute of Nuclear Energy Research, Atomic Energy Council, Taoyuan 32546, Taiwan; ^3^Yokohama City University Graduate School of Medicine, Yokohama 236-0004, Japan; ^4^Small Animal MRI, Biomedical Research Imaging Center, University of North Carolina at Chapel Hill, Chapel Hill, NC 27599, USA

Molecular nuclear imaging agents enable the comprehensive characterization of therapeutic intervention and can be used in patient selection, pharmacokinetic, dosage-finding, and proof-of-concept studies. The effort in image-guided cell therapy and theranostic approaches in parallel with instrumentation development would be more comprehensive in the outcome assessment of patient response to treatment. To extend the threshold of being able to provide personalized therapy for patients, the integration of imaging findings to genomic and proteomic systems profiling is essential to validate targeted pathways.

The Food and Drug Administration (FDA) permits radiopharmaceuticals produced under well-controlled conditions in central commercial facilities to be distributed to local clinics where they are administered. In addition, radiopharmaceutical production process must adhere to Current Good Manufacturing Practice (CGMP) compliance to ensure the quality of drug product that meets acceptance criteria. The CGMP compliance covers manufacturing process and facility, quality guidelines, and personnel training. Y.-T. Chi et al. reported the design of CGMP production for ^18^F- and ^68^Ga-radiopharmaceuticals. The Pharmaceutical Inspection Convention and Pharmaceutical Inspection Cooperation Scheme (jointly referred to as PIC/S) are two international instruments between countries and pharmaceutical inspection authorities, which provide together an active and constructive cooperation in the field of Good Manufacturing Practice (GMP). PIC/S' mission is to lead the international development, implementation, and maintenance of harmonized CGMP standards and quality systems of inspectorates in the field of medicinal products. They reviewed FDA and PIC/S guidelines for the synthesis of radiopharmaceuticals. Two examples, ^68^Gallium-[1,4,7,10-tetraazacyclododecane-N,N′,N′′,N′′′-tetraacetic acid]-D-Phe^1^,Tyr^3^-octreotate (^68^Ga-DOTATATE) and ^18^F-fluorodeoxyglucose (^18^F-FDG), were manufactured under CGMP process. They have reviewed acceptance criteria for these clinic useful radiopharmaceuticals.

Radiopharmaceutical chemistry requires intricate handling of radioactive materials, fast reaction times, ease of synthesis, and reproducible results. In the preclinical setting, radiopharmaceuticals are typically synthesized manually. Such applications use* in vitro* and small animal models to validate the agent and require low levels of radioactivity. The use of manual synthesis for clinical imaging, however, is challenging for multiple reasons: (1) clinical agents must meet strict sterility and pyrogenicity requirements which are validated from batch to batch; (2) batch-to-batch reproducibility is required to demonstrate suitable radiochemical yield, radiochemical purity, and other quality control analyses; (3) synthesis time must be fast when dealing with radionuclides with a short half-life; (4) clinical studies require multiple patient doses and would expose radiochemists to much higher levels of radioactivity; and (5) production cost and availability of the technology may limit the viability of the agent in routine clinical practice. I.-H. Shih et al. reported the manufacturing of a cGMP grade of [^18^F]fluoropropoxytryptophan (^18^F-FTP) to assess tryptophan transporters. PET imaging studies were performed with ^18^F-FTP and ^18^F-FDG in prostate and small cell lung tumor-bearing animal models. They have reported that ^18^F-FTP could be synthesized with high radiochemical yield. They conclude that ^18^F-FTP may provide potential applications in differential diagnosis and prediction of early treatment response for carcinoids.

Molecular imaging science has been focused on imaging guidance in the areas of targeting epigenetic abnormalities and disease microenvironment in overcoming resistance in diseases. Multimodality imaging using noncytotoxic triple fusion (TF) reporter genes is an important application for cell-based tracking, drug screening, and therapy. Y.-J. Hsieh et al. reported the rational design of a triple reporter gene for multimodality molecular imaging. They reported that an optimized triple fusion reporter constructed with DsRedm-fl-ttksr39 was developed and validated for more effective and sensitive* in vivo* animal imaging using fluorescence, bioluminescence, and PET imaging modalities. Their findings may facilitate different fields of biomedical research and applications.

The use of image-guided technologies to select patient for personalized therapy and to monitor therapeutic outcomes is the focus of this special issue. S. H.-H. Yeh et al. reported the evaluation of inhibitory effect of recreational drugs on dopaminergic terminal neuron by ^18^F-FDOPA and whole-body autoradiography. They used ^18^F-FDOPA PET imaging and autoradiographic techniques toassess the impact of recreational drugs such as ketamine, cocaine, and methamphetamine on dopamine neurons in peripheral organs. They have demonstrated that both dynamic ^18^F-FDOPA PET and autoradiography were useful crossing-validating tools to evaluate the alteration of dopaminergic neurons in peripheral tissues. D. Smith et al. reported the application of patched targeting peptides for imaging and treatment of hedgehog positive breast tumors. Hedgehog (Hh) signaling is involved in breast cancer growth and metastasis and high tumor Sonic Hedgehog (SHh) expression is correlated with poor prognosis in invasive ductal carcinoma. Peptides which bind the PTCH receptor have recently been reported to have a growth inhibitory effect in tumors with activated Hh signaling. These peptides may be used as molecular imaging probes to monitor changes in Hh expression after chemotherapy. Their studies showed that peptides which bind the SHh docking site in PTCH-1 correlate with PTCH-1 expression and can be used to image PTCH-1* in vivo*. They conclude that radiolabeled peptides may enable examining the activity of the Hh signaling pathway and evaluating response to anticancer therapies.

In summary, this special issue covers advances in molecular imaging in drug manufacturing under CGMP environment, preclinical drug discovery, image-guided drug response, and target validation using PET/SPECT hybrid with CT.

*David J. Yang* *Lan Pham* *Mei-Hsiu Liao* *Fan-Lin Kong* *Hiroji Uemura* *Yen-Yu Ian Shih*

